# Light Harvesting Nanoprobe for Trace Detection of Hg^2+^ in Water

**DOI:** 10.3390/molecules28041633

**Published:** 2023-02-08

**Authors:** Aleksandr Chepak, Denis Balatskiy, Mikhail Tutov, Aleksandr Mironenko, Svetlana Bratskaya

**Affiliations:** 1 Institute of Chemistry, Far Eastern Branch, Russian Academy of Sciences, 159, Prosp. 100-letiya Vladivostoka, Vladivostok 690022, Russia; 2 Department of Chemistry and Materials, Institute of High Technologies and Advanced Materials, Far Eastern Federal University, 10 Ajax Bay, Russky Island, Vladivostok 690922, Russia

**Keywords:** fluorescent nanoprobe, Hg, sensing, FRET

## Abstract

The continuously increasing flow of toxic heavy metals to the environment due to intensive industrial activity and tightening requirements with regard to the content of metal ions in drinking and discharged waters urges the development of affordable and sensitive devices to the field control of pollutants. Here, we report a new thiated Rhodamine-lactam probe for Hg^2+^ detection and demonstrate how its sensitivity can be increased via the incorporation of the probe molecules into the optically transparent siloxane-acrylate coatings on polymethyl methacrylate and, alternatively, into the water-dispersible light-harvesting FRET nanoparticles (NPs), in which dye cations are separated by fluorinated tetraphenylborate anions. We have shown that the optimization of the FRET NPs composition had allowed it to reach the antenna effect of ~300 and fabricate “off/on” sensor for Hg^2+^ ion determination in aqueous solutions with the detection limit of ~100 pM, which is far below the maximum permissible concentration (MPC) of mercury in drinking water recommended by the World Health Organization. Although this work is more proof-of-concept than a ready-to-use analytical procedure, the suggested approaches to fabrication of the FRET NPs based on the popular rhodamine-lactam platform can be used as a background for the development of low-cost portable sensing devices for the extra-laboratory determination of hazardous metal ions.

## 1. Introduction

Drinking water quality and safety is a highly sensitive issue for the population, even in developed countries with high standards of water treatment technologies. As a result of industrial activities, continuously increasing flow of wastewaters containing toxic heavy metals, which are already harmful to human health at ppb level, it is released into the environment. Moreover, the synergetic effects of several pollutants, especially the combination of toxic metals with antibiotics and the low efficacy of water treatment toward new classes of emerging pollutants, make on-site water quality monitoring extremally important. Although analytical methods are available for the majority of metal ions (Hg, Cd, Pb, As), the fabrication of highly sensitive and cost-effective devices is still a challenge. According to a World Health Organization (WHO) report [1], there are no extra-laboratory methods for determining mercury at the level of maximum permissible concentration (MPC). Lead and cadmium in the field can only be determined at levels close to the MPC. Despite the high sensitivity of electrochemical sensors described in the literature, the detection limit for Hg achieved to date is close to the MPC for mercury in water [2]. Microfluidic devices using optical sensors are characterized by a limit of detection (LOD), which is at least tenfold higher than the MPC values in drinking water recommended by WHO for the most toxic metals: Hg [3] and Pb [4]. Alternative ultrasensitive techniques such as surface-enhanced Raman scattering [5,6,7,8] or surface-enhanced fluorescence [9,10,11,12] allow for the determination of significantly lower analyte concentrations; however, they require powerful and expensive equipment, both for measurements and for the fabrication of nanostructured enhancing substrates, which limits the use of such sensors devices for monitoring water quality in problematic regions, as in the active gold mining and processing industry, for example.

To date, the most traditional and widely used detection method is based on the application of fluorescent probes, which have become an effective analytical tool due to the unique capability for sensitive monitoring of metal ions [13,14], anions [15,16], reactive oxygen species [17,18], or biomolecules [19,20]. With these benefits, the sensitivity of such fluorescent probes is strictly limited by their brightness (the product of absorption and the photoluminescence quantum yield). Thus, one of the brightest probes, which is based on the Rhodamine 6G scaffold [21,22], has binding constants ~10^3^–10^5^ that are similar to other types of fluorophores [23]; a molar absorptivity of ~10^5^, and show a typical working range of sensor operation from ~1μm to ~0.1 mM. 

To improve the performance of fluorescent probes, it is necessary to enhance the response signal significantly via the incorporation into the hydrophobic environment [24] or by pumping the probe with a much brighter quantum emitter via Förster resonance energy transfer (FRET). However, the required bright emitters are usually large nanoparticles (NPs) which are inefficient FRET donors, since their sizes are beyond the FRET radius (1–10 nm) [19]. An alternative approach based on pumping the probe with multiple molecular emitters was recently demonstrated in light-harvesting FRET NPs, in which dye cations are separated by fluorinated tetraphenylborate anions that prevent dye self-quenching [25,26,27,28]. In these NPs, a short inter-fluorophore distance controlled by the counterion enables ultrafast dye–dye excitation energy migration on a femtosecond time scale through the whole particle within the fluorescence lifetime until it reaches a donor close to the acceptor leading to the FRET. Therefore, the energy can be transferred beyond the Förster radius from multiple donors to a single acceptor, providing a basis for signal amplification [19]. In a recent work, we adopted this light harvesting approach to pump the probe for the determination of Cu^2+^ ions, which demonstrated a ~100 fold decrease of LOD [29]. Here, we demonstrate the further development of this method. Using a specially designed novel thiated Rhodamine-lactam probe for mercury detection, we have reoptimized the light harvesting system based on protonated Coumarin-30 (further referred to as **C30**) cation and sodium tetrakis [3,5-bis(1,1,1,3,3,3-hexafluoro-2-methoxy-2-propyl)phenyl] borate anion (further referred to as **F12**). As a result, an ultrasensitive “off/on” nanoprobe with an antenna effect of 300 and an impressive Hg^2+^ LOD of ~100 pM was obtained.

## 2. Results and Discussion

**Figure 1:**
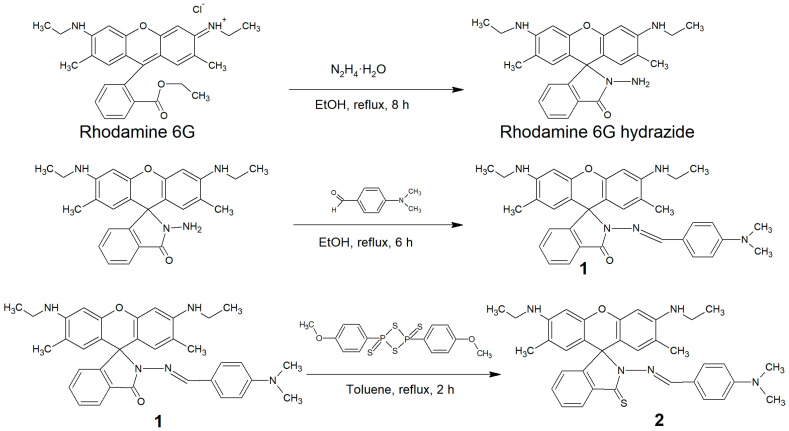
The synthesis route of d114 (compound **2**).

**Figure 2:**
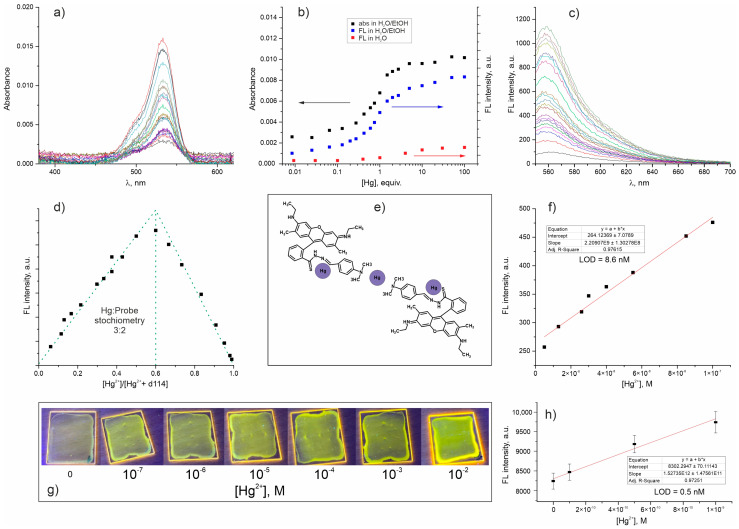
Characterization of **d114** probe in H_2_O/C_2_H_5_OH (1/1, *v*/*v*): the change of absorbance (**a**,**b**) and fluorescence (**c**,**b**) spectra upon successive addition of Hg^2+^ to d114 solution with concentration of 10^−6^ M; Job’s plots (method of continuous variation) showing formation of 3:2 **Hg^2+^/d114** complexes in solution (**d**); proposed binding scheme between **d114** and Hg^2+^ ions (**e**); dependence of the response signal on the concentration of the analyte and results of the Hg^2+^ LOD calculations (**f**). Photo of PMMA slides with latex coatings containing **d114** probe immersed for 5 min in Hg^2+^ solutions (pH = 6) and excited with 365 nm LED (**g**); and results of the Hg^2+^ LOD calculations for d114-doped coating (**h**).

The development of an ultra-sensitive nanoprobe for detection of trace amounts of mercury was started from the design of a suitable molecular probe. The probe designed for Hg^2+^ detection (**d114**) is a derivative of **d98** probe obtained by substitution amide oxygen with sulfur (Figure 1). We expected that **d98** and **d114** would be a pair of probes for Cu^2+^ and Hg^2+^ detection with properties similar to those of the already known rhodamine hydrazide and thiohydrazide probes. However, the characterization of **d114** in solution revealed that its optical response has a fundamentally different character. In sharp contrast to **d98**, which forms a colored and non-luminescent 1:1 complex with Cu^2+^ [29], the successive addition of Hg^2+^ to **d114** solution results in a simultaneous increase in absorption (Figure 2a,b) and luminescence (Figure 2b,c). The method of continuous variation revealed the formation of 3:2 **Hg^2+^/d114** complex (Figure 2d,e), whose binding constant in H_2_O/C_2_H_5_OH (1/1, *v*/*v*), calculated as described in [30], was equal to 2.8·10^7^ (Figure S3, Supplementary information). This value is higher than those usually reported for Rhodamine-based probes for metal ions [21,22] and other probes for Hg^2+^ [3]. 

The LOD for mercury with **d114** in H_2_O/C_2_H_5_OH (1/1, *v*/*v*) was 8.6 nM or 1.72 µg/L (Figure 2f) that is below mercury MPC in drinking water recommended by WOS (6 µg/L) [1] and the maximum concentration level (MCL) for the discharge to the aquatic environment regulated by US Environmental Protection Agency (2 µg/L) [31]. This makes the **d114** probe superior to most of the reported optical sensors. However, the intensity of the **d114** optical signal is solvent-dependent, as is often observed for most fluorophores [32]; it is much lower in water than it is in an H_2_O/C_2_H_5_OH mixture (Figure 2b) or in pure organic solvents, so the analyzed sample has to be diluted first. This increases LOD and somehow complicates express analysis if this probe is used in the devices for the field application. 

In order to estimate the potential of **d114** for the modification of channels in microfluidic sensors, we fabricated transparent latex coatings with a thickness of ~600 nm on PMMA slides (Figure 2g), which have demonstrated a fast response time in aqueous solutions with an LOD of ~0.5 nM or 0.1 µg/L (Figure 2h). Sensitivity enhancement of the probe after immobilization in the coatings can be related to the metal ions preconcentration on the carboxylic group of the siloxane-acrylate latex, which was earlier demonstrated by our group [33]. Furthermore, despite a hydrophobicity sufficient to prevent fluorophore release to the aqueous solution and the high stability of the siloxane-acrylate coatings in aqueous solutions at pH < 11, they were permeable for metal ions [34,35]. A large excess of carboxylic groups in the coating did not interfere with the sensory response due to the much higher binding constant of Hg^2^ with S-containing ligand (**d114**). Thus, the Hg^2+^ LOD reached using **d114**-doped siloxane-acrylate coatings was sufficient for application in extra-laboratory portable devices to control the mercury concentration in water below MPC [1] and MCL [31]. 

However, field monitoring of mercury concentrations in the areas of concern, e.g., water reservoirs in highly developed industrial areas and gold mining and processing sites requires more sensitive methods of detection due to the fast accumulation of mercury by living organisms and organic matter and its chronic toxic effects. Thus, the next step consisted in development of light-harvesting FRET NPs, which was done to reach Hg^2+^ LOD in water at the level far below ppb. 

Our previous work [29] was focused on finding the conditions for the formation of the brightest coumarin-based NPs, which then were doped with a Cu^2+^-probe (**d98**) to yield a nanoprobe with FRET enhancement. In order to reach a high luminescence quantum yield of such NPs, they had to be assembled at a high counterion/dye ratio in the solution. However, at a large excess of counterion (**F12**) in water, amines and their derivatives (including lactams, i.e., all rhodamine-based probes) are protonated and associated into insoluble NPs. This makes the enhancing of the sensory response of classical rhodamine-based probes impossible, since “switching ON” the probe inside the NPs is determined by the NPs component (**F12** counterion) rather than by the analyte (target ion). To overcome this limitation, we have modified a Cu^2+^-sensitive probe (**d98**) with an electron-donating amino aromatic group, which was introduced to change the optical response of the probe via photoinduced electron transfer (PET) fluorescence quenching that allowed Cu^2+^ detection via the quenching of the initially bright nanoparticles. Thus, **d98** has two binding sites—hydrazone and amino aromatic fragments (see compound **1** structure in Figure 1). The hydrazone fragment has affinity for both Cu^2+^ and H^+^ ions, while the amino aromatic one only has an affinity for H^+^. When **d98** was associated with an excess of F12 in acidic solution or inside sensitive NPs, both binding sites of the molecule were occupied by H^+^ ions, so the probe existed in colored and fluorescent form, which was efficiently pumped by the light harvesting matrix, since the emission band of Coumarin 30 significantly overlaps with excitation band of **d98**. The binding of the Cu^2+^ ion to **d98** either dissolved or incorporated in NPs leads to the displacement of both protons from the probe molecule and a decrease in fluorescence intensity. Thus, the concentration of Cu^2+^ ions can be determined from the change in the fluorescence intensity of the solution at constant absorption (i.e., by decrease in FLQY).

Taking into account the stoichiometry of binding and the bright luminescence of the **Hg^2+^/d114** complex, the approach suggested for Cu^2+^ determination using FRET NPs has to be modified. To obtain a system capable of modulating the **d114** sensory response under the action of an ion analyte, it was necessary to reduce the excess of the counterion in NPs in order to minimize its effect on the **d114** binding sites responsible for sensing properties, and thus maintain the ability of the probe to interact with the analyte. 

For this, **Coumarin-30/F12** (further referred to as **C30/F12**) nanoparticles doped with **d114** were obtained at a **C30/F12** ratio of 1:2. We showed earlier that **C30/F12** NPs obtained at pH = 5 in sodium-acetate buffer solution have low colloidal stability. Here we have used Millipore ® water with a neutral pH that greatly improved the colloidal stability of the obtained solutions. According to the dynamic light scattering data, the size of the obtained NPs was ~250 nm, while the size of unstable particles obtained at the same ratio of precursors in the sodium acetate buffer was ~130 nm.

**Figure 3:**
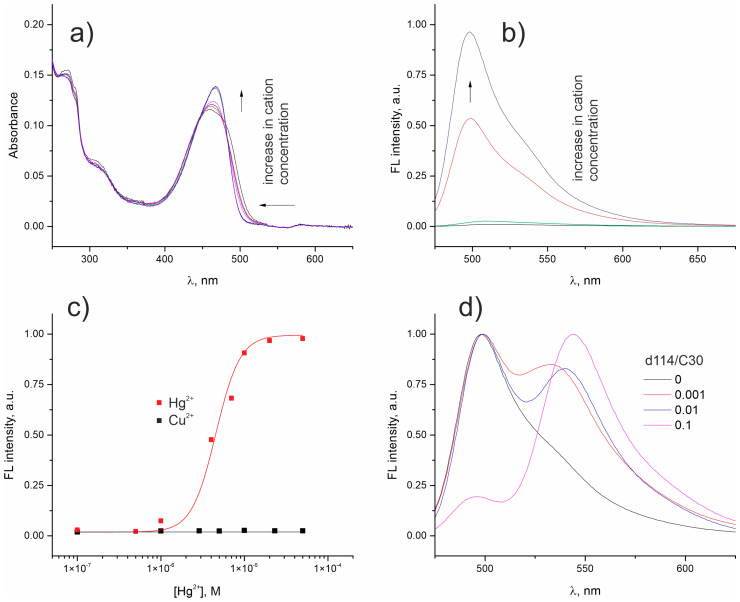
The change in absorption (**a**) and luminescence (**b**) spectra of **C30/F12** 1/2 NPs in aqueous solution (pH = 6) upon addition of Hg^2+^. The dependence of luminescence intensity of **C30/F12** 1/2 NPs on Hg^2+^ and Cu^2+^ concentration (**c**). The luminescence spectra of **C30/F12** NPs doped with different amount of **d114** (**d**).

Since colloidal solutions, and especially ionic associates, are quite sensitive to changes in the ionic composition of the solution, we first tested pure **C30/F12** NPs solution by titration with Hg^2+^ and Cu^2+^, i.e., cations with a high affinity to **d114**, to determine the concentration range over which **C30/F12** NPs are stable. Figure 3 shows that at Hg^2+^ concentrations up to 10^−6^ M, the intensity of the luminescence of the **C30/F12** NPs solution virtually did not change, while at higher concentrations a significant increase in luminescence intensity and a slight shift of the absorbance band was observed (Figure 3a–c). Next, the corresponding solutions of **C30/F12** NPs doped with different amounts of **d114** were obtained and tested by the addition of Hg^2+^ at a concentration of 10^−6^ M to establish the maximum **C30/d114** ratio at which FRET from coumarin nanoantenna to **d114** is observed (Figure 3d). The estimated FRET efficiency in NPs with the composition **d114/C30/F12** of 0.001/1/2 was ~30%, which means that the antenna effect in the system is about 300. This value is rather close to that (1000) reported by the Klymchenko group [25]. 

**Figure 4:**
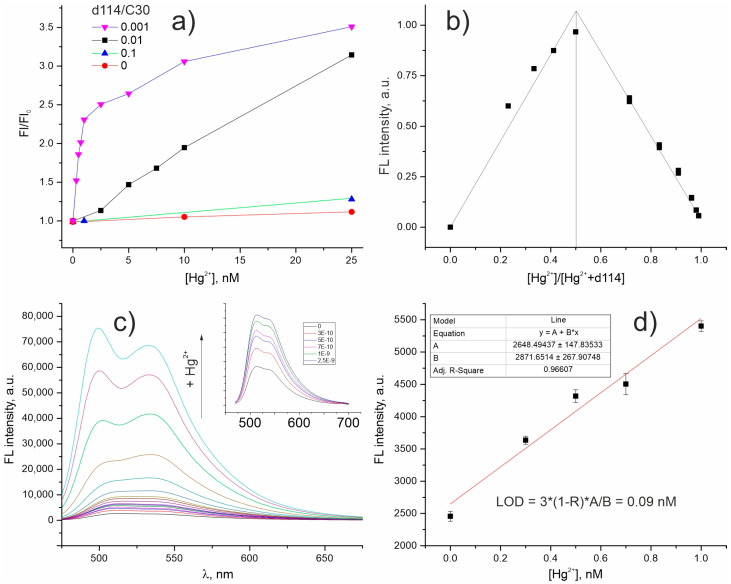
Characterization of **d114/C30/F12** NPs in aqueous solution (pH = 6): dependence of luminescence intensity change (F/F_0_) on Hg^2+^ concentration for NPs with different **d114** content (**a**); Job’s plots showing 1:1 interaction stoichiometry of **d114** and Hg^2+^ inside light-harvesting NPs **d114/C30/F12** 0.001/1/2 (**b**); the change in the luminescence spectrum of **d114/C30/F12** 0.001/1/2 solution upon the addition of Hg^2+^(the inset shows the spectra in Hg^2+^ concentration range up to 2.5 nM) (**c**); dependence of the response signal on the analyte concentration in the range up to 1nM (**d**).

The titration of **d114/C30/F12** NPs in aqueous solution with Hg^2+^ ions have shown that at a lower **d114** content in the system, a sharper change in the signal intensity in the region of low concentrations is observed (Figure 4a). Additionally, the method of continuous variation was applied for **d114/C30/F12** 0.001/1/2 NPs to analyze binding stoichiometry between **d114** and Hg^2+^ and demonstrated inflection at a 1:1 ratio of **Hg/d114** (Figure 4b). The change in the signal intensity (Figure 4c) in the Hg^2+^ concentration range up to 1 equiv. of d114 was approximately linear (Figure 4a,c); the Hg^2+^ LOD calculated for nanoprobe **d114/C30/F12** 0.001/1/2 according to signal-to-noise ratio of 3 was 0.09 nM (Figure 4d). It is also worthy of note that, similarly to Cu^2+^-sensitive FRET NPs doped with **d98** [29], the presence of low amounts (at least up to 10^–6^ M) of interfering ions, including Cu^2+^, does not significantly affect the sensitivity of mercury detection (Figure S4, Supplementary Information).

## 3. Materials and Methods

### 3.1. Chemicals and Instruments

Rhodamine 6G (99%, Sigma Aldrich, St. Louis, MO, USA), Coumarin 30 (99%, Sigma Aldrich), 4-(dimethylamino)benzaldehyde (99%, Sigma Aldrich), Lawesson reagent 2,4-Bis-(4-methoxyphenyl)-1,3-dithia-2,4-diphosphetane 2,4-disulfide (97%, Sigma Aldrich), hexane (95%, Sigma Aldrich), chloroform (99%, Sigma Aldrich), ethyl acetate (99.8%, Sigma Aldrich), dichloromethane (99%, Sigma Aldrich), hydrazine monohydrate (98%, Sigma Aldrich), sodium tetrakis [3,5-bis(1,1,1,3,3,3-hexafluoro-2-methoxy-2-propyl)phenyl]borate trihydrate (99%, Sigma Aldrich), and silica gel (100/200 μm) were used as received. Siloxane-acrylate latex (KE 13-36) dispersion with a solid phase content of 46%, were produced by the Scientific Production Association “Astrokhim” (Elektrostal’, Moscow Region, Russia). All other reagents were of analytical grade and were used without purification. All aqueous solutions were prepared using Millipore ^®^ water.

The Fourier transform infrared radiation (FT-IR) spectra of the compounds in the range 400–4000 cm^−1^ were recorded using a Perkin Elmer Spectrum 100BX II spectrometer in KBr pellets. ^1^H, ^13^C NMR spectra were performed on a Bruker Avance 400 with the frequency of proton resonance of 400 MHz using CDCl_3_ as the solvent and tetramethylsiliane as the internal reference. Mass spectrometry was performed on a Shimadzu LCMS-2010 LC-ESI/MS system. The UV-Vis spectra were obtained using a Shimadzu UV-2600 spectrophotometer equipped with a Shimadzu ISR 2600 Plus integrating sphere. The fluorescence spectra were obtained with a Shimadzu RF-6000 spectrofluorophotometer with a 1 cm standard quartz cell. For selectivity experiments, freshly prepared stock solutions of the nitrate salts of Hg^2+^, Cu^2+^, Ni^2+^, Mg^2+^, Al^3+^, Zn^3+^, Co^2+^, Ag^+^ in Millipore ^®^ water were used. All of the titration experiments were recorded at room temperature. The size of fluorescent NPs was determined using a ZetaSizer Nano ZS analyzer (Malvern Instruments Ltd., UK). The analyzed solutions were filtered through a 0.8 µm syringe filter to remove dust particles, while the optical absorption of the solutions was monitored using UV-visible spectroscopy before and after filtration to ensure that the NPs were not retained by the membrane. The measurements were carried out in automatic operation mode at room temperature. The pH measurements were carried out using a Sartorius Professional Meter PP-50. The thickness of the sensing coatings was measured using an Auto SE spectroscopic ellipsometer (Horiba, Japan).

### 3.2. Synthesis and Characterization of ***d114***

**Rhodamine 6G hydrazide** was synthesized using the similar procedure as described in [36] with minor modifications. Briefly, 300 mg, (6.3 · 10^−4^ mol) of rhodamine 6G and hydrazine monohydrate (1.0 mL, 1.9·10^−2^ mol) were dissolved in 20 mL of ethanol (95%). The mixture was refluxed for 12 h. The solvent was removed under the reduced pressure and the crude product was purified by flash column chromatography on a silica gel with dichloromethane/ethyl acetate (*v*/*v* = 4/1) as the eluent to afford the product as a crystal powder (220 mg, Yield 80%). If a larger amount of the substance needs to be synthesized, purification can be carried out by recrystallization from ethanol. ^1^H NMR (400 MHz, CDCl_3_, ppm, δ): 7.96 (m, 1H), 7.45 (m, 2H), 7.06 (m, 1H), 6.39 (s, 2H), 6.26 (s, 2H), 3.58 (s, 2H), 3.54 (br.s, 2H), 3.22 (q, 4H), 1.92 (s, 6H), 1.32 (t, 6H); ^13^C NMR (100 MHz, CDCl_3_, ppm, δ): 14.38, 16.34, 37.97, 65.66, 96.42, 104.47, 117.60, 122.65, 123.42, 127.31, 127.75, 129.45, 132.22, 147.15, 151.35, 151.83, 165.82; ESI-MS (*m*/*z*, +νe mode) 529.63 [M + H]^+^, calc. for C_26_H_29_N_4_O_2_^+^ is 529.22; Elemental Analysis data: Calc. C, 72.87; H, 6.59; N, 13.07; Expt. C, 72.97; H, 6.66; N, 12.89.

**Compound 1 (d98)** was synthesized as described in our previous work [29]. An amount of 300 mg (7 · 10^−4^ mol) of rhodamine 6G hydrazide and 220 mg (1.4 · 10^−3^ mol) of 4-(dimethylamino)benzaldehyde were dissolved in 15 mL of ethanol (95%). The mixture was refluxed for 6 h. The solvent was removed under reduced pressure and the crude product was purified by flash column chromatography on a silica gel with hexane/ethyl acetate (*v*/*v* = 2/1) as the eluent to afford the product as a crystal powder (290 mg, Yield 74%). ^1^H NMR (400 MHz, CDCl_3_, ppm, δ): 8.33 (s, 1H, H(24)), 8.03–8.04 (m, 1H, H(12)), 7.06–7.08 (m, 1H, H(9)), 7.45 (s, 1H, H(18)), 7.47 (s, 1H, H(19)), 7.61 (s, 1H, H(15)), 7.59 (s, 1H, H(22)), 7.47–7.49 (m, 2H, H(10), H(11)), 6.42 (br. s, 2H, H(26), H(30)), 6.40 (br. s, 2H, H(27), H(29)), 3.50 (br.s, 2H, H(33), H(36)), 3.21–3.26 (q, 4H, H(34), H(37)), 2.96 (s, 6H, H(41), H(42)), 1.89 (s, 6H, H(32), H(39)), 1.34–1.36 (t, 6H, H(35), H(38)); ^13^C NMR (100 MHz, CDCl_3_, ppm, δ): 14.77, 16.68, 22.70, 29.70, 31.94, 38.37, 40.23, 59.19, 65.72, 76.70, 77.03, 77.34, 96.78, 106.63, 111.51, 117.94, 123.21, 123.51, 127.76, 128.98, 133.00, 147.44, 151.17, 152.40, 164.83; ESI-MS (*m*/*z*, +νe mode) 560.81 [M + H]^+^, calc. for C_35_H_38_N_5_O_2_^+^ is 560.30; Elemental Analysis data: Calc. C, 75.11; H, 6.66; N, 12.51; Expt. C, 75.43; H, 6.70; N, 12.42.

**Compound 2 (d114).** The scheme of synthesis is presented in Figure 1. An amount of 30 mg (5.37 · 10^−4^ mol) of **1** and 22 mg (5.37 · 10^−4^ mol) of Lawesson reagent were mixed in 15 mL of absolute toluene. The mixture was refluxed for 1 h. An additional portion of Lawesson reagent (11 mg, 2.69 · 10^−4^ mol) was then added to the reaction mixture, and then the mixture was refluxed for 1 h. The solvent was removed under reduced pressure and the crude product was purified by flash column chromatography on a silica gel with hexane/ethyl acetate (*v*/*v* = 1/1) as the eluent to afford the product as a red powder (17.7 mg, 57%). ^1^H NMR (400 MHz, CDCl_3_, ppm, δ): 8.53 (s, 1H, H(24)), 8.12–8.14 (m, 1H, H(12)), 7.05–7.07 (m, 1H, H(9)), 7.37 (s, 1H, H(18)), 7.40 (s, 1H, H(19)), 7.66 (s, 1H, H(15)), 7.69 (s, 1H, H(22)), 7.40 (m, 2H, H(10), H(11)), 6.30 (br. s, 2H, H(26), H(30)), 6.62 (br. s, 2H, H(27), H(29)), 3.50 (br.s, 2H, H(33), H(36)), 3.20–3.21 (q, 4H, H(34), H(37)), 3.01 (s, 6H, H(41), H(42)), 1.92 (s, 6H, H(32), H(39)), 1.29–1.33 (t, 6H, H(35), H(38)); ^13^C NMR (100 MHz, CDCl_3_, ppm, δ): 14.36, 14.92, 17.02, 22.92, 29.93, 32.15, 38.78, 40.38, 96.75, 111.76, 118.32, 122.32, 127.29, 127.91, 130.39, 130.64, 132.12, 150.20, 152.39, 155.67, 159.72; ESI-MS (*m*/*z*, +νe mode) 576.34 [M + H]^+^, calc. for C_35_H_38_N_5_OS^+^ is 576.28; Elemental Analysis data: Calc. for C_35_H_37_N_5_OS: C, 73.01; H, 6.48; N, 12.16; S, 5.57; Expt. C, 73.33; H, 6.54; N, 12.18; S, 5.41. Mp: 208–210 °C (with decomposition). ^1^H NMR and ^13^C NMR spectra are shown in Figures S1 and S2, respectively (Supplementary information).

### 3.3. Fabrication of the Sensing Coating Containing ***d114*** on PMMA

Transparent sensing coatings were fabricated by casting 0.156 mL of siloxane-acrylate latex dispersion in H_2_O/CH_3_OH (1/1, *v*/*v*) with solid content of 2% and **d114** concentration of 10^−5^ M on the polymethyl methacrylate (PMMA) slides with the surface area of 4.05 cm^2^. Coatings were left for drying in the air at T = 25 °C for 24 h before use. 

### 3.4. Preparation of Fluorescent NPs

1 mM of **Coumarin-30** (further referred to as **C30**) in CH_3_CN, 1–100 µM of **d114** in CH_3_CN and 1mM of sodium tetrakis [3,5-bis(1,1,1,3,3,3-hexafluoro-2-methoxy-2-propyl)phenyl]borate) (further referred to as **F12**) in CH_3_CN were used as stock solutions. Typically, 10 µL of **C30**, 0–10 µL of **d114** and 20 µL of **F12** stock solutions were mixed in a plastic vial and quickly added to 5 mL of deionized water under intensive stirring to yield a colloidal solution of fluorescent NPs. The resulting colloidal solutions were left for equilibration for 60 min before use.

## 4. Conclusions

Very strict limitations to the mercury content in discharge and drinking water results in the failure of the most fluorescent probes to provide the required detection limit. It is therefore necessary to enhance the response signal significantly so that it can be realized, for example, via pumping the probe with a much brighter quantum emitter via Förster resonance energy transfer (FRET). Pursuing the goal of developing optical sensors for the extra-laboratory determination of mercury at ultralow concentrations, we have designed a new thiated Rhodamine derivative (**d114**) with a luminescence response inverted from “on/off” to “off/on”, which demonstrated an Hg ^2+^ LOD of 8.6 nM in H_2_O/C_2_H_5_OH (1/1, *v*/*v*) solution and of 0.5 nM in water when used for doping transparent siloxane acrylate coating on PMMA. 

To further improve the sensitivity of this fluorophore, we have modified our earlier developed strategy for the fabrication of Cu^2+^—sensitive probe-doped light-harvesting FRET nanoparticles (NPs) formed by Coumarin-30 **(C30)** and sodium tetrakis [3,5-bis(1,1,1,3,3,3-hexafluoro-2-methoxy-2-propyl)phenyl]borate) (**F12**). Due to the different nature of the sensory response and binding stoichiometry of the earlier developed probe for Cu^2+^ ions and **d114** for Hg^2+^ ions, the composition of the FRET NPs has to be optimized. We have investigated the dependence of the fluorescence signal on the **d114/C30** mol ratio and shown that **a** nanoprobe with the composition of **d114/C30/F12** 0.001/1/2 provided an antenna effect of ~300. Taking advantage of the FRET effect, ~100-fold decrease of mercury LOD was reached using a simple spectrofluorometer. Thus, the demonstrated approach makes possible the detection of extremely toxic Hg^2+^ cations in water at concentrations far below the maximum permissible concentration (MPC) in drinking water set by the World Health Organization and can be possibly extended to other optical sensors based on the Rhodamine-lactam platform.

## Data Availability

Not applicable.

## Supplementary Materials

The following supporting information can be downloaded at: https://www.mdpi.com/article/10.3390/molecules28041633/s1, Figure S1: ^1^H NMR spectrum of **d114** recorded in CDCl_3_; Figure S2: ^13^C NMR spectrum of **d114**; Figure S3: Fluorescence intensity of **d114** solution versus free ligand concentration, [free Hg^2+^] calculated according to the law of mass action; Figure S4: Influence of the interfering metal ions (10^−6^ M) on response value of **d114/C30/F12** 0.001/1/2 NPs in the presence of 10^−8^ M Hg^2+^.
