# Supplementary material for: Light Harvesting Nanoprobe for Trace Detection of Hg2+ in Water
Source: Molecules. 2023 Feb 8;28(4):1633. doi: 10.3390/molecules28041633 (PMC9965521; doi:10.3390/molecules28041633)
Supplement: Supplementary file 1 [file molecules-28-01633-s001.zip › molecules-2163641-supplementary.pdf]

Supplementary materials

Light Harvesting Nanoprobe for Trace Detection of Hg^{2+} in Water

Aleksandr Chepak [1], Denis Balatskiy [1], Mikhail Tutov [1,2], Aleksandr Mironenko [1] and Svetlana Bratskaya [1,*]

1 Institute of Chemistry, Far Eastern Branch, Russian Academy of Sciences, 159, Prosp. 100-letiya Vladivostoka, Vladivostok 690022, Russia
2 Far Eastern Federal University, 10 Ajax Bay, Russky Island, Vladivostok 690922, Russia
* Correspondence: s.bratskaya@gmail.com

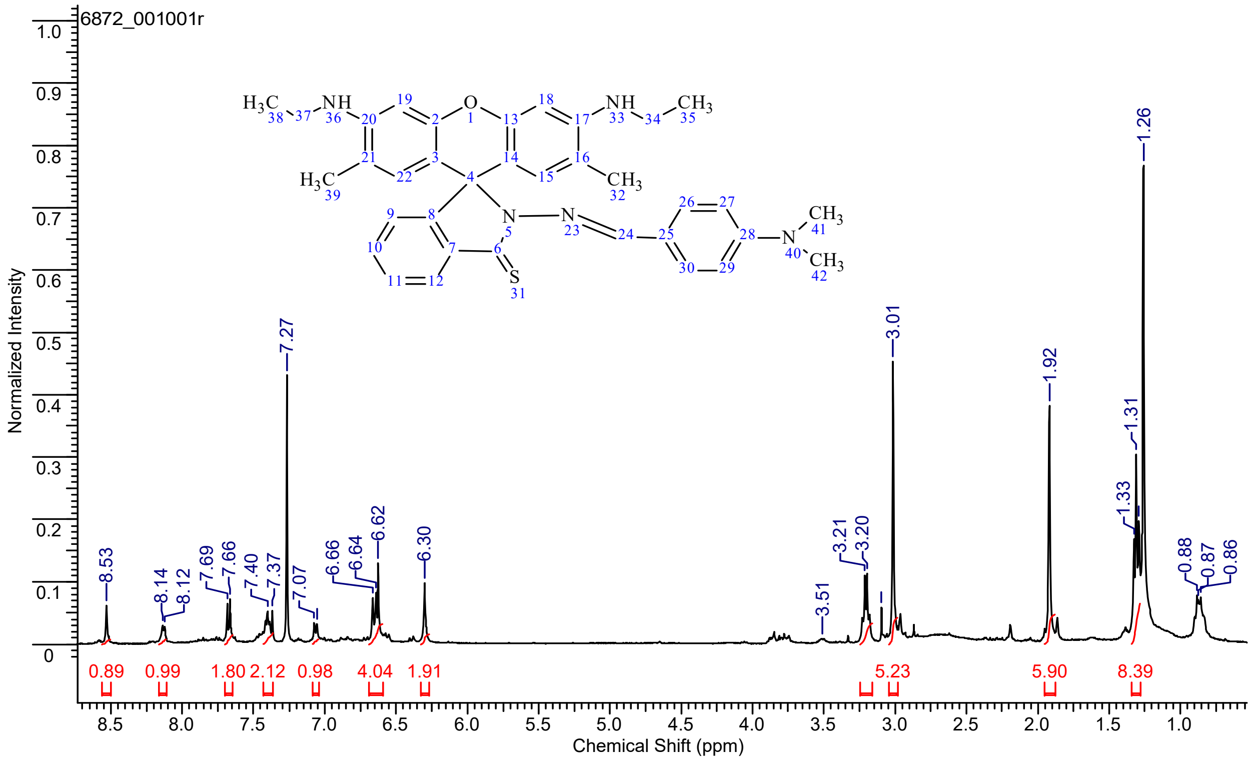

Figure S1. ^{1}H NMR spectrum of d114 recorded in $CDCl_3$.

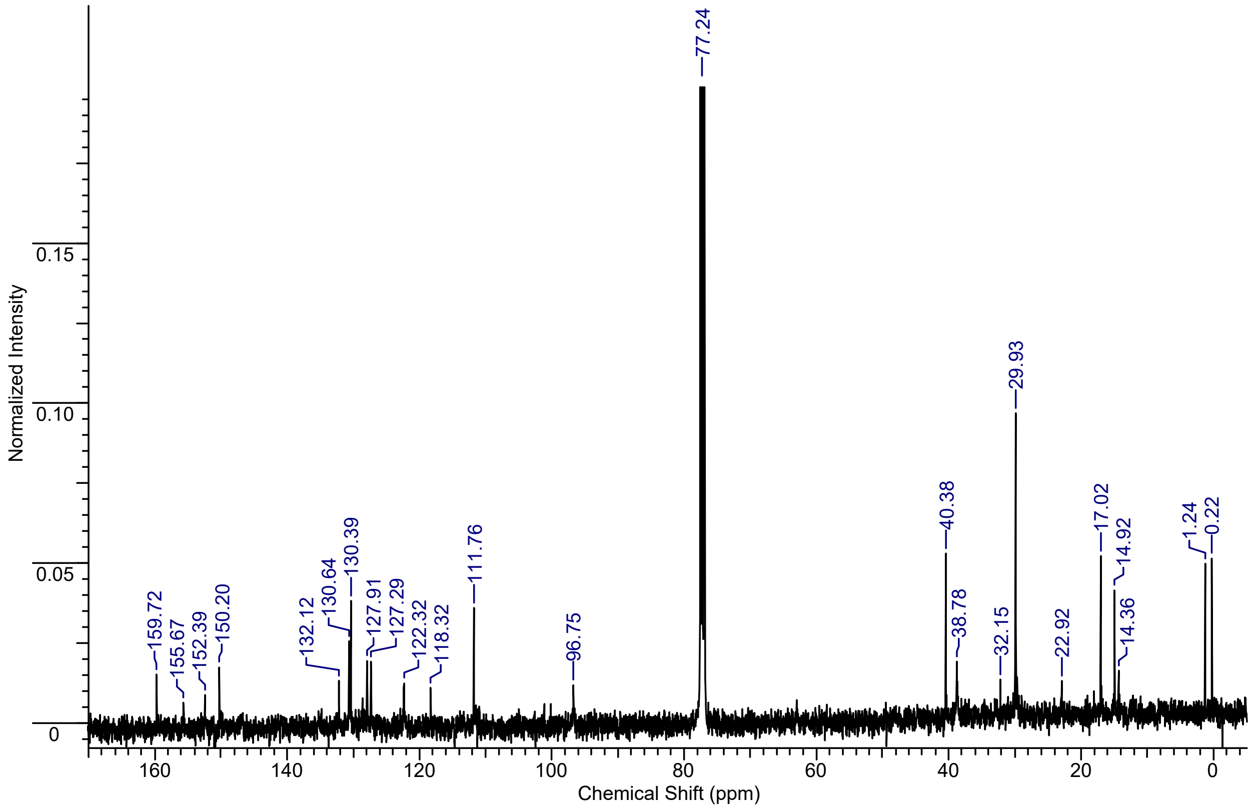

Figure S2. ^{13}C NMR spectrum of d114 recorded in $CDCl_3$.

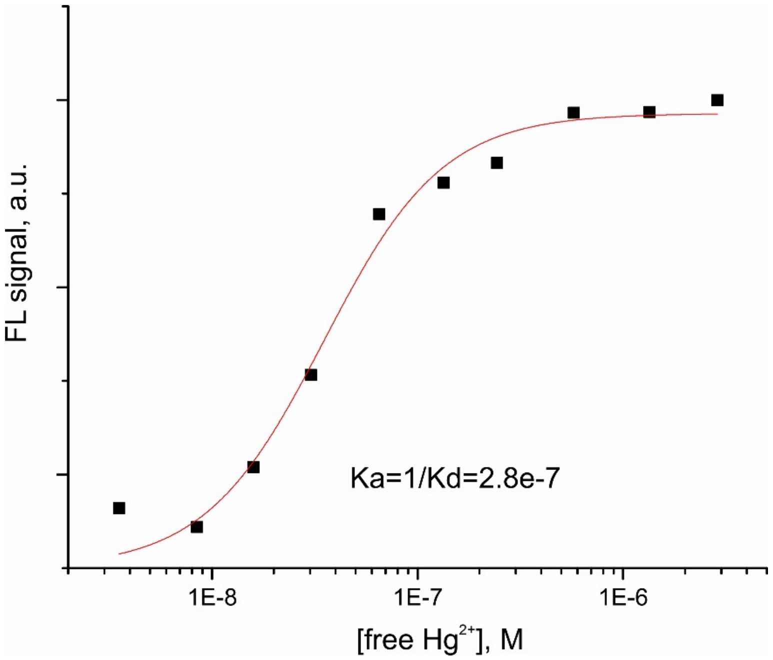

Figure S3. Fluorescence intensity of d114 solution versus free ligand concentration, [free Hg^{2+}] calculated according to the law of mass action.

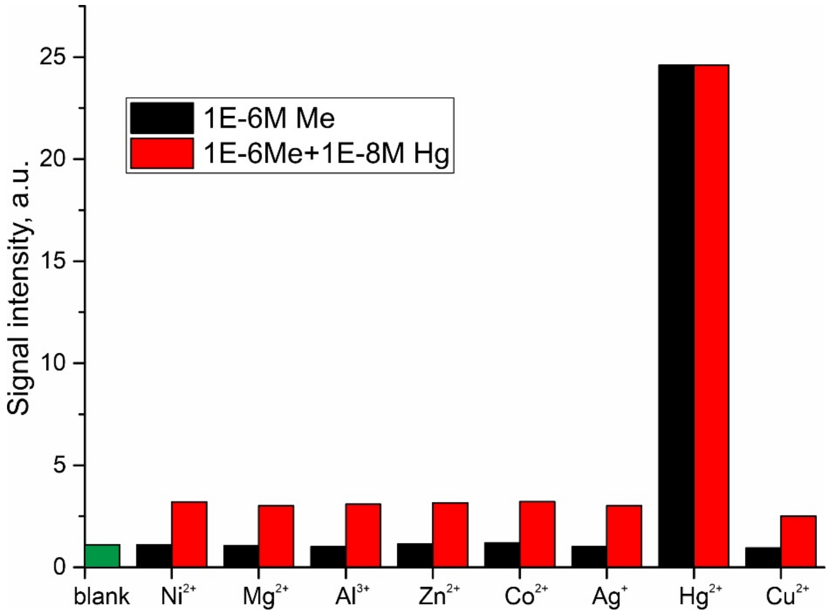

Figure S4. Influence of the interfering metal ions (10^{-6} M) on response value of **d114/C30/F12** 0.001/1/2 NPs in the presence of 10^{-8} M Hg^{2+}
